# Homogeneous Cytochrome 579 Is an Octamer That Reacts Too Slowly With Soluble Iron to Be the Initial Iron Oxidase in the Respiratory Chain of *Leptospirillum ferriphilum*

**DOI:** 10.3389/fmicb.2021.673066

**Published:** 2021-05-03

**Authors:** Robert C. Blake, John E. Shively, Russell Timkovich, Richard Allen White

**Affiliations:** ^1^Division of Basic Pharmaceutical Sciences, Xavier University of Louisiana, New Orleans, LA, United States; ^2^Division of Immunology, Beckman Research Institute of the City of Hope, Duarte, CA, United States; ^3^Department of Chemistry, University of Alabama, Tuscaloosa, AL, United States; ^4^Department of Bioinformatics and Genomics, University of North Carolina, Charlotte, NC, United States; ^5^Department of Bioinformatics and Genomics, University of North Carolina, Kannapolis, NC, United States; ^6^Australian Centre for Astrobiology, University of New South Wales, Sydney, NSW, Australia

**Keywords:** *Leptospirillum*, cytochrome 579, electron transport, aerobic respiration, cooperative kinetics

## Abstract

The exact role that cytochrome 579 plays in the aerobic iron respiratory chain of *Leptospirillum ferriphilum* is unclear. This paper presents genomic, structural, and kinetic data on the cytochrome 579 purified from cell-free extracts of *L. ferriphilum* cultured on soluble iron. Electrospray mass spectrometry of electrophoretically homogeneous cytochrome 579 yielded two principal peaks at 16,015 and 16,141 Daltons. N-terminal amino acid sequencing of the purified protein yielded data that were used to determine the following: there are seven homologs of cytochrome 579; each homolog possesses the CXXCH heme-binding motif found in *c*-type cytochromes; each of the seven sequenced strains of *L. ferriphilum* expresses only two of the seven homologs of the cytochrome; and each homolog contains an N-terminal signal peptide that directs the mature protein to an extra-cytoplasmic location. Static light scattering and macroion mobility measurements on native cytochrome 579 yielded masses of 125 and 135 kDaltons, respectively. The reduced alkaline pyridine hemochromogen spectrum of the purified cytochrome had an *alpha* absorbance maximum at 567 nm, a property not exhibited by any known heme group. The iron-dependent reduction and oxidation of the octameric cytochrome exhibited positively cooperative kinetic behavior with apparent Hill coefficients of 5.0 and 3.7, respectively, when the purified protein was mixed with mM concentrations of soluble iron. Consequently, the extrapolated rates of reduction at sub-mM iron concentrations were far too slow for cytochrome 579 to be the initial iron oxidase in the aerobic respiratory chain of *L. ferriphilum*. Rather, these observations support the hypothesis that the acid-stable cytochrome 579 is a periplasmic conduit of electrons from initial iron oxidation in the outer membrane of this Gram-negative bacterium to a terminal oxidase in the plasma membrane.

## Introduction

*Leptospirillum ferrooxidans* (Markosyan, 1972; Hippe, 2000), *Leptospirillum ferriphilum* (Coram and Rawlings, 2002), and *Leptospirillum ferrodiazotrophum* (Tyson et al., 2005) are species of mesophilic, vibrioid, or spiral-shaped Gram-negative prokaryotes that can be acquired as pure cultures from national/commercial culture collections. Other potential species in that genus, *Leptospirillum rubarum* (Goltsman et al., 2009), *Leptospirillum thermoferrooxidans* (Golovacheva et al., 1992; Hippe, 2000), and a group designated as “*Leptospirillum* group IV UBA BS” (Goltsman et al., 2013), have been reported, but none of these strains is generally available at the present time. All of these organisms are obligately acidophilic chemoautotrophs that are only known to respire aerobically on reduced soluble iron or ferrous iron-containing sulfide minerals to obtain biochemical energy, making them among the most metabolically restricted organisms known. Despite these restrictions, these microorganisms are widely distributed in both pristine and industrial bioleaching environments, where they are frequently thought to be the critical biological catalysts in the metal sulfide biooxidation process (Sand et al., 1992; Rawlings et al., 1999; Rawlings and Johnson, 2007; Garcia-Moyano et al., 2008).

The identities and cellular locations of the respiratory chain components that are responsible for iron oxidation in the *Leptospirilli* are poorly understood. Prior reductionist studies reported that cell-free extracts derived from iron-grown *L. ferrooxidans* (Hart et al., 1991), *L. ferriphilum* (Ram et al., 2005), or a microbial biofilm community with a low diversity of microbes that was dominated by *Leptospirillum* group II bacteria (Singer et al., 2008) all contained readily discernible quantities of an acid-stable, acid-soluble cytochrome with an unusual absorbance maximum at 579 nm in the reduced state. Although the likely location of this acid-compatible cytochrome 579 was in the acidic periplasm, incomplete reduction of the protein was noted using 30 mM Fe(II) at pH 2.0. Another novel cytochrome with a unique reduced absorbance maximum at 572 nm was purified from the same biofilm communities (Jeans et al., 2008). Cytochrome 572, which was reported to be localized in the outer membrane, was readily and completely reduced by soluble Fe(II) at low pH. These observations led to the working hypothesis that cytochrome 572 was the initial iron oxidase in the outer membrane, while cytochrome 579 served to shuttle electrons from the outer membrane across the periplasm to a terminal oxidase in the plasma membrane. However, the same group also noted that the reactivity of cytochrome 579 was very dependent on the developmental stage of the biofilm (Singer et al., 2010). Thus, cytochrome 579 isolated from early-stage biofilms was much more reactive with Fe(II) at low pH than was cytochrome 579 isolated from late-stage biofilms, an observation that was consistent with the hypothesis that cytochrome 579 might function as an alternative iron oxidase in early developmental-stage biofilms.

This paper describes selected structural and functional properties of cytochrome 579 that was purified to electrophoretic homogeneity from cell-free extracts of the type strain of *L. ferriphilum*. Although this cytochrome possessed the signature absorbance maximum at 579 nm in the reduced state, it nonetheless differed in a number of ways from the purified cytochromes 579 introduced above. First, the reduced alkaline pyridine hemochromogen spectrum of this protein was different from that of the cytochrome 579 purified from the biofilm community (Singer et al., 2008). Second, this cytochrome appeared to function as an octamer in its native state, while the purified cytochromes 579 from both *L. ferrooxidans* (Hart et al., 1991) and the microbial biofilm (Singer et al., 2008) appeared to be monomeric in their native states. Last, but not least, the observed pseudo-first order rate constants for the iron-dependent reduction and oxidation of the purified cytochrome exhibited extraordinary positive cooperativity, a property not reported for the other cytochromes 579. Thus, while the rates of reduction of this cytochrome were sufficiently rapid *in vitro* to be physiologically significant at the high ferrous concentrations achieved in the laboratory, the corresponding rates of reduction were far too slow to be significant at the concentrations of soluble iron likely to be encountered by *L. ferriphilum* in its natural habitat.

## Materials and Methods

### Cell Culture and Quantification of Bacteria

*Leptospirillum ferriphilum* DSMZ 14647 was cultured autotrophically on soluble ferrous ions at 35°C in the medium described elsewhere (Tuovinen and Kelly, 1973) adjusted to pH 1.5 and amended with 44 g/L of FeSO_4_∙7H_2_O. Larger quantities of bacteria for protein purification purposes were cultured in an apparatus that permitted the *in situ* electrochemical reduction of the product ferric ions to achieve enhanced yields of the bacteria (Blake et al., 1994). Cells were cultured on a continuous basis in a 45-liter vessel under an applied current of 30 A with a voltage that varied from 4 to 7 V. When suspensions of cells were removed for further experimental purposes, an equal volume of fresh medium was added back to the culture vessel. Cells thus obtained were harvested by centrifugation, washed three times with 0.02 M H_2_SO_4_, pH 1.5, and resuspended in sufficient 0.02 M H_2_SO_4_ to achieve a stock suspension of 1.5 × 10^10^ cells per ml. Each stock suspension was stored at 4°C for up to 2 weeks while structural or spectroscopic experiments were conducted on aliquots of the cells.

Bacterial growth was monitored by quantifying bacterial carbon with a Dohrmann DC-190 high temperature carbon analyzer (Rosemount Analytical, Inc., Santa Clara, CA, United States). The premise was that any carbon detected in solutions of inorganic media at pH 1.5 must be proportional to the biomass accumulated by the obligate autotrophs that thrived on that media. Bacterial, or cell-associated, carbon was taken as the difference between the total organic carbon in the cell suspension minus that remaining after the sample had been filtered through a 0.2 μm pore-size filter.

Absolute numbers of *L. ferriphilum* cells were determined by electrical impedance measurements on a Multisizer 4 particle counter (Beckman Coulter, Inc., Brea, CA, United States) fitted with a 30-μm aperture. The instrument was programmed to siphon 50 μl of sample that contained Isoton II as the electrolyte. The current applied across the aperture was 600 μA. Voltage pulses attendant with impedance changes as particles passed through the aperture were monitored with an instrument gain of 4.

### Purification of Red Cytochrome

The purification of cytochrome 579 from cell-free extracts of *L. ferriphilum* cultured on soluble iron was conducted as described elsewhere (Ram et al., 2005). Briefly, cells were resuspended in 0.01 N sulfuric acid (4 ml/g wet cell paste) and subsequently disrupted by sonic oscillation for 1 min/g wet cell paste at a power output of 125 watts. Care was taken to maintain the temperature of the solution below 7°C. Centrifugation of the sonicate at 10,000 × *g* for 10 min yielded a red pellet and a cloudy red supernatant. The pellet was resuspended in 0.01 N sulfuric acid (1.5 ml/g original wet cell paste) and centrifuged as described above; the two supernatants were then combined.

The combined supernatants were then subjected to ammonium sulfate precipitation. The bulk of the red cytochrome precipitated between 45 and 95% saturated ammonium sulfate. A red pellet was obtained by centrifugation of the 95%-saturated solution for 20 min at 20,000 × *g*. The pellet was dissolved in and dialyzed against 0.01 N sodium acetate buffer, pH 5.5. The preparation of red cytochrome was then applied to a column of carboxymethylcellulose equilibrated and developed with the same acetate buffer. All of the colored cytochrome eluted from the column in the void volume. A curious feature of this purification procedure was that the cytochrome that eluted in the void volume of the carboxymethylcellulose column was always observed to be in the yellow-green reduced state, regardless of the oxidation state of the protein when it was applied to the column. The source and identity of the reducing equivalents were not determined. The reduced protein thus obtained was converted back to the red oxidized state after mixing with a few grains of ferric sulfate. The red cytochrome at this point exhibited a single band when analyzed by standard SDS-PAGE. SDS-PAGE under reducing conditions was routinely performed on a Pharmacia Phast™ system using PhastGel gradient 10–15 polyacrylamide gels and PhastGel SDS buffer strips; proteins within the gels were visualized using a standard Coomassie Blue stain. Proteins in solution were quantified using the copper-bicinchoninic acid assay (Smith et al., 1985). The purified red protein was concentrated to approximately 10 mg protein/ml by ultrafiltration through an Amicon Centriprep-30 membrane concentrator and stored at 4°C in 0.001 N sulfuric acid. The red cytochrome remained fully redox-active for at least 6 months under these storage conditions.

### Protein Subunit Structural Characterization

Amino terminal microsequencing of the purified red cytochrome was accomplished as described previously (Ronk et al., 1991). BLASTp and tBLASTn sequence similarity searches using default parameters were conducted on non-redundant protein sequences and RefSeq Genome databases, respectively, maintained for those purposes by the NCBI (Claverie and Notredame, 2007). *Leptospirillum ferriphilum* strains and their corresponding RefSeq assembly accession numbers were as follows: DSMZ 14647, GCF_000755505.1; ML-04, GCF_000299235.1; YSK, GCF_000695975.1; Sp-Cl, GCF_001280545.1; DX, GCF_002002505.1; ZJ, GCF_002002665.1; pb_238, GCF_900198525.1; and CF-1, GCF_001186405.1.

The subunit molecular weight of the purified protein was obtained by liquid chromatography coupled with electrospray mass spectrometry. The protein sample (1.0 μl, 14 pmoles) was injected onto a capillary reversed phase HPLC column (25 mm ID, Vydac C18) developed at 2 μl/min with a linear gradient from 22% A (0.1% trifluoroacetic acid) to 82% B (trifluoroacetic acid/water/acetonitrile, 0.1/9.9/90, v/v/v) over 30 min. The absorbance at 200 nm was monitored on an ATI/Kastos UV detector. The sample was mixed with 2-methoxyethanol (2 μl/min) for introduction into the electrospray on a Finnigan TSQ700 triple quadrupole mass spectrometer.

### Native Protein Structural Characterization

The apparent molecular mass of the native cytochrome in solution was measured by two means. The first means involved Rayleigh light scattering analyses on a Dawn DSP laser photometer equipped with multiple detectors (Wyatt Technology Corp., Santa Barbara, CA, United States). The instrument was run in the batch mode with 10 ml of cytochrome sample per measurement. The protein concentrations in these static light scattering measurements ranged from 1.15 to 5.77 μg or purified protein per milliliter.

The second means involved macroion mobility measurements of the purified protein using a model 3980C macroIMS macroion mobility spectrometer from TSI, Inc. (Shoreview, MN, United States). The instrument consisted of a model 3480 electrospray aerosol generator, a model 3080C electrostatic classifier, and a model 3776 macroion/nanoparticle detector. Samples of 320 ng protein/ml were prepared by diluting the 10 mg protein/ml stock solution into 20 mM ammonium acetate. Any nonvolatile salts that were present in the stock protein preparations were sufficiently diluted in the resulting dilute samples such that any spurious nonprotein peaks that were observed at low molecular masses did not interfere with the interpretation of the protein spectra. Control analyses using purified protein that had been extensively dialyzed against 20 mM ammonium acetate demonstrated that neither the apparent mass nor the shape of the resulting protein peak in the macroIMS spectrum were changed by the desalting procedure. The ammonium acetate was prepared fresh weekly and passed through a 0.2 μm filter to remove any traces of particulate matter.

### Spectral and Kinetic Characterization of Purified Cytochrome 579

Absorbance spectra were obtained on a Cary 14 dual-beam spectrophotometer as described earlier (Blake and Shute, 1987). The alkaline pyridine hemochromogen spectra of cytochrome 579 were obtained as described elsewhere (Bartsch, 1971).

Kinetic measurements were performed on an RSM-1000™ stopped flow spectrophotometer equipped with a rapid-scan module that permitted the collection and analysis of absorbance data as a function of both time and wavelength (Blake and Shute, 1987). The samples of cytochrome 579 and ferrous or ferric ions were prepared in identical solutions of sulfuric acid, pH 1.5, and added to separate syringes of the stopped flow spectrophotometer. The temperature of the driving syringes was maintained at 35 ± 1°C by circulating water; room-temperature solutions were allowed to equilibrate for 15 min in the driving syringes. Reactions were initiated by rapidly mixing 0.14 ml of the solution from each driving syringe. An operational bandwidth of 1.0 nm provided acceptable signal to noise characteristics between 350 and 700 nm.

Global fits of absorbance changes as a function of both time and wavelength over the 230-nm range of 370–600 nm were accomplished by the singular value decomposition method (DeSa and Matheson, 2004) using analysis software provided by On Line Instrument Systems, Inc., Bogart, GA, United States. The output of applying the singular value decomposition method to each three-dimensional data set of absorbance as a function of time and wavelength generated three products as follows: (i) a matrix of spectral eigenvectors that represented the changes in absorbance for the principal absorbing species; (ii) a matrix of kinetic eigenvectors that represented the mechanism that accounted for the time dependencies of the principal absorbance changes; and (iii) a diagonal matrix of eigenvalues that minimized the differences between the calculated and the observed data.

### Phylogenetic Analysis

The alignment of different amino acid sequences was conducted using the MAFFT multiple sequence alignment software (v7.271) with option local pair and maximum iterations of 1,000 (Katoh and Standley, 2013). Maximum likelihood phylogenetic trees were constructed using the IQ-TREE stochastic algorithm (1.6.2; Nguyen et al., 2014). Model selection was conducted with the ModelFinder selection method (Kalyaanamoorthy et al., 2017) with a total of 1,000 boot-strap replicates using UFBoot2 (Hoang et al., 2017) and visualized with SeaView4 (Gouy et al., 2009). *Candidatus Manganitrophus noduliformans* (WP_168058047.1) was used as an outgroup for cytochrome 579.

## Results

### Cell Culture by Electrochemical Reduction

A principal practical barrier to fundamental or applied research on *L. ferriphilum* is simply achieving sufficient cell mass for meaningful studies. Efforts to harvest bacteria cultured on insoluble substrates such as pyrite are hampered by the inability to readily separate the cell mass from the particulate inorganic materials, either insoluble substrates that remain or insoluble products such as jarosite. Efforts to cultivate the same bacteria on soluble ferrous ions at acidic pH are hampered by the discouragingly poor cell yield achieved by typical batch culture. A typical culture of *L. ferriphilum* yields roughly 10–20 mg wet cell paste per liter of acid ferrous sulfate medium. Greatly enhanced yields of *L. ferriphilum* cultured autotrophically on soluble ferrous ions were achieved for these studies by the *in situ* electrochemical reduction of soluble ferric ions using an apparatus described previously (Blake et al., 1994).

**Figure 1:**
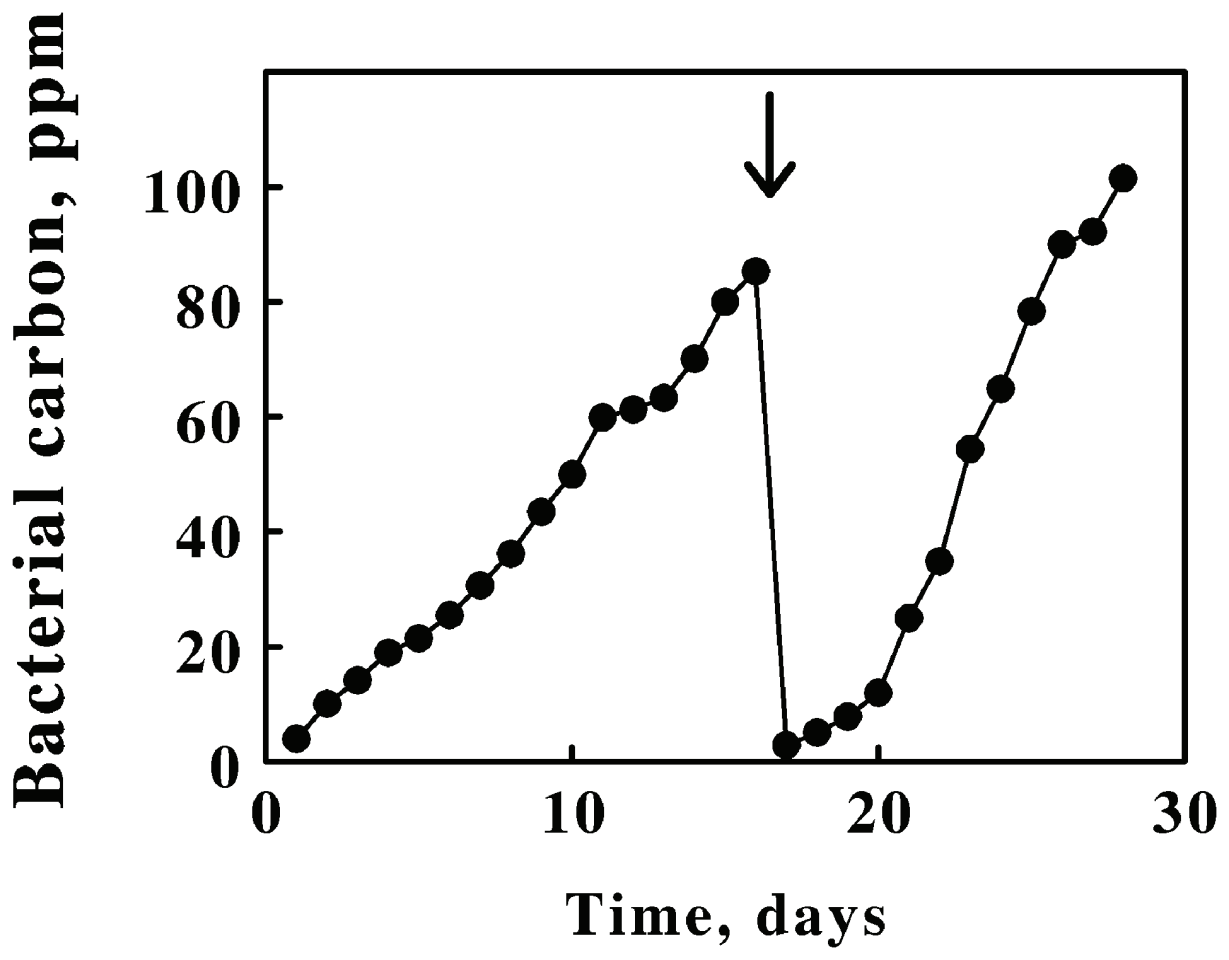
Enhanced yields of *Leptospirillum ferriphilum* achieved by *in situ* electrochemical reduction of soluble iron in the growth medium. Zero time was taken as the start of electrochemical reduction after the culture had already achieved stationary phase on soluble iron in the absence of reduction. The position of the *bold arrow* corresponds to the replacement of 95% of the cell suspension with fresh growth medium.

Figure 1 shows the accumulation of bacterial carbon as a function of time in electrolyzed ferrous sulfate media inoculated with *L. ferriphilum*. Electrolytic reduction was initiated when the batch culture had achieved stationary phase (the 0,0 intercept in Figure 1). By reducing the product ferric ions back to the ferrous state at the cathode, the bacteria in the catholyte experienced an endless supply of the growth substrate and continued to prosper until some other factor in the growth medium became limiting. A notable feature of electrolytic growth was the apparent linear, not exponential, dependence of bacterial growth with time. Such linear rates suggest that bacterial growth was limited by some factor that was introduced in a linear fashion. Because the electrical current necessary to maintain a steady state level of iron reduction increased significantly as the cell numbers increased over the time course of each culture, the most likely growth-limiting candidate was either oxygen or carbon dioxide. In any event, every 100 ppm of bacterial carbon was observed to correspond to approximately 1.3 g wet cell paste/liter medium. Thus, 45 L of electrolyzed medium yielded nearly 60 g of the iron-grown *L. ferriphilum* approximately every 2 weeks.

### Structural Properties of the Cytochrome 579 Polypeptide

**Figure 2:**
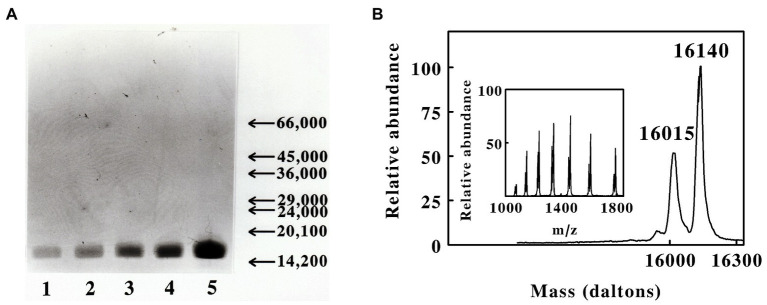
Masses of the polypeptides present in the preparation of cytochrome 579 purified from cell-free extracts of *L. ferriphilum*. **(A)** Sodium dodecyl sulfate-polyacrylamide gel electrophoresis of the purified cytochrome under denaturing and reducing conditions. Protein bands were visualized by staining with Coomassie Blue. *Lanes 1* through *5* represent 0.85, 1.7, 3.3, 7.5, and 15 μg of purified protein, respectively. The *arrows* and *numbers* on the ordinate represent the migration positions and masses, respectively, of stained bands associated with protein molecular mass standards (primary data not shown). The mass standards from high to low were bovine serum albumin, ovalbumin, yeast alcohol dehydrogenase, carbonic anhydrase, bovine chymotrypsin, soybean trypsin inhibitor, and bovine *α*-lacalbumin. **(B)** Deconvoluted positive-ion electrospray mass spectrum of purified cytochrome 579, showing the masses of the neutral polypeptides. *Inset*, primary data of the electrospray mass spectrum of the purified protein, showing the ions formed by multiple attachment of protons to the molecules.

SDS-PAGE conducted under reducing conditions revealed that the purified protein was comprised of a single polypeptide with an apparent molecular mass of around 16,000 Daltons. Figure 2A shows SDS-PAGE analyses conducted on five different concentrations of the purified red cytochrome. The purified protein appeared to be a single, albeit somewhat diffuse, band at all concentrations, with no visible evidence of contaminating proteins as revealed by staining with Coomassie Blue.

Initial evidence for structural heterogeneity in the purified cytochrome 579 came from liquid chromatography coupled with electrospray mass spectrometry. Figure 2B shows the positive-ion electrospray mass spectrum of cytochrome 579. The main figure shows the deconvoluted spectrum for the neutral cytochrome(s) obtained using operating and analysis software provided with the mass spectrometer. The principal mass peaks observed at 16,015 and 16,140 Daltons may represent two distinct but nearly identical proteins. Alternatively, the small mass difference could derive from missing amino- or carboxyl-terminal amino acids due to proteolysis activity during preparation or from post-translational modifications of part of the sample.

**Figure 3:**
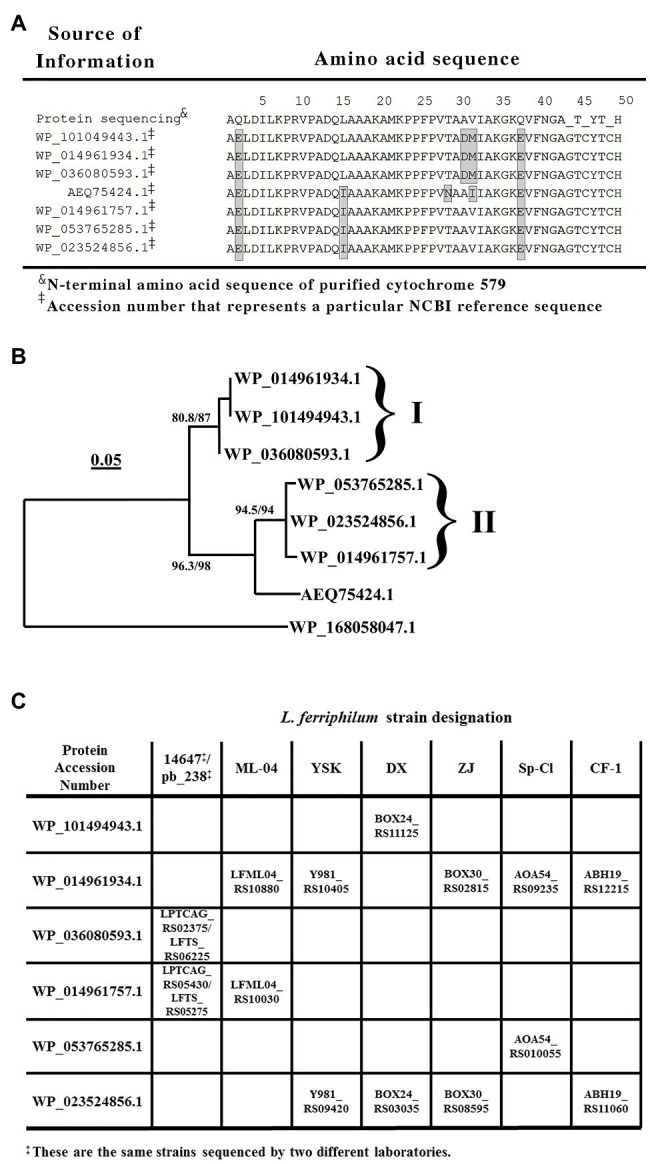
Sequence comparisons of cytochromes 579 that could be expressed in different strains of *L. ferriphilum*. **(A)** Alignment of amino acid sequences obtained from either the N-terminal amino acid sequencing of cytochrome 579 purified from the type strain of *L. ferriphilum* or from tBLASTn searches of the genomes of purified strains of *L. ferriphilum* that are contained in the NCBI database. The amino acids within each shaded rectangle are those that differ from the corresponding amino acids determined in the protein sequencing data presented herein. Beyond amino acid number 40, the individual amino acid peaks in each round of the sequencing became sufficiently small and irregular so as to prevent a confident identification of the amino acid released during each round of degradation. **(B)** A rooted phylogenetic tree based on seven cytochrome 579 amino acid sequences taken from the proteins represented by the accession numbers shown in **(A)**. The outlying sequence (lowermost item shown in the figure) represents a monoheme *c*-type cytochrome of similar length present in the genomic sequence of *Candidatus Manganitrophus noduliformans*. The tree was inferred using the maximum likelihood method with 1,000 replicates. The *brackets and Roman numerals* identify the two groups of cytochromes 579 that possess different N-terminal signal peptide sequences. **(C)** Identities and genetic loci of the two proteins in each strain of *L. ferriphilum* that bear a high level of sequence homology with the cytochrome 579 purified from the type strain of *L. ferriphilum*. The genetic loci within each rectangle were obtained from the tBLASTn searches conducted on each strain.

The uppermost line of text in Figure 3A shows the N-terminal amino acid sequence of purified cytochrome 579 as obtained by automated Edman degradation performed on a gas-phase microsequencer. The amino acid chromatograms produced at each cycle of the automated degradation reactions were all consistent with the hypothesis that cytochrome 579 was comprised of a single type of polypeptide chain. That is, each individual chromatogram exhibited one major peak corresponding to the derivatized amino acid released from the polypeptide during that cycle. However, microsequencing analyses performed on internal peptides derived from enzymatic or chemical cleavage of cytochrome 579 also yielded sporadic instances where individual amino acid chromatograms were observed with two major peaks of approximately equal intensity (primary data not shown). These 2-peak chromatograms were frequently interspersed among more routine 1-peak chromatograms generated from the same HPLC-purified peptide(s). One hypothesis that is consistent with these observations was that the purified cytochrome 579 was comprised of two or more different polypeptide chains that were highly homologous, but not quite identical. This apparent heterogeneity in the primary structure of purified cytochrome 579 made the determination of the primary structure by traditional amino terminal microsequencing activities highly unlikely.

The N-terminal sequence of 49 amino acids was used as the query sequence in BLASTp searches of the *L. ferriphilum* species (taxid 178,606) contained in the NCBI non-redundant protein database (Figure 3A). These protein-protein searches yielded the sequences of seven different proteins that exhibited a high degree of homology to the query sequence. These seven amino acid sequences are aligned in their entirety in Supplementary Figure S1. Each of the seven proteins contained an N-terminal segment of 19–23 amino acids that was tentatively identified as a signal peptide (Ivankov et al., 2013) that targeted the mature protein to the periplasm of this Gram-negative eubacterium. The corresponding N-terminal sequences that remained after the removal of the respective signal peptides are those represented by the seven remaining N-terminal sequences shown in Figure 3A. The homology among these amino acid sequence segments is evident. One other noteworthy feature of these sequence comparisons is that every mature protein in Figure 3A possesses the CXXCH heme-binding motif (between amino acids 45 and 49) that is the only generally recognized signature sequence that is common to all *c*-type cytochromes.

Figure 3B shows a rooted phylogenetic tree based on eight complete protein sequences, seven homologs of cytochrome 579 and an outgroup protein comprised of a *c*-type cytochrome present in the genome of *Candidatus Manganitrophus noduliformans*. Six of the seven sequences fell into one of the two groups associated within the two *brackets* or clades in Figure 3B that were arbitrarily designated with the Roman numerals *I* and *II*. The three proteins within *clade I* share the same signal peptide sequence, which is different from the signal peptide sequence shared by the three proteins within *clade II*. The protein represented by the accession number AEQ75424.1 has the same signal peptide sequence as those proteins within *clade II*. However, the rest of its primary structure is sufficiently different so as to not warrant its inclusion within either of the two clades. It should be noted that the sequence for this latter protein was derived from a community-wide sequencing project, not from a purified strain of *L. ferriphilum*.

The results of BLASTp searches do not permit one to identify which homologous protein(s) may actually be expressed by individual strains of the same species. Rather, the BLASTp algorithm searches the relevant database for all proteins of all assemblies of the single species, in this case *L. ferriphilum*. It seemed unlikely that every individual strain of *L. ferriphilum* contains the genetic material to express all seven of the highly homologous forms of cytochrome 579 that are present in the species. To determine which cytochromes 579 could be expressed in individual strains of *L. ferriphilum*, translating tBLASTn searches were conducted on the nucleotide assemblies of the seven strains of *L. ferriphilum* that are currently available (Mi et al., 2011; Cardenas et al., 2014; Jiang et al., 2015; Ferrer et al., 2016; Issotta et al., 2016; Zhang et al., 2017; Christel et al., 2018). These searches were limited to those nucleotide assemblies derived from the genomic sequencing of pure strains; no relevant metagenomic sequence data were considered.

The data in Figure 3C show the results of conducting tBLASTn searches on each of the seven strains of *L. ferriphilum* that are available in the NCBI database using six of the seven proteins in Figures 3A,B as the queries. Each strain of *L. ferriphilum* contains the genetic material to express two, and only two, of the seven homologs of cytochrome 579. The corresponding genetic locus for each homolog in each strain is identified in the figure. Further, one of the two genes in each strain codes for a protein contained within one clade in Figure 3B, while the other of the two genes codes for a protein contained within the other clade in Figure 3B. That is, each strain possesses the genetic codes to express one protein from the contents of each of the two clades in Figure 3B. Genomic sequences for the homolog represented by the accession number AEQ75424.1 were not identified in any of these seven strains using the tBLASTn searches.

### Structural Properties of the Native Cytochrome 579

**Figure 4:**
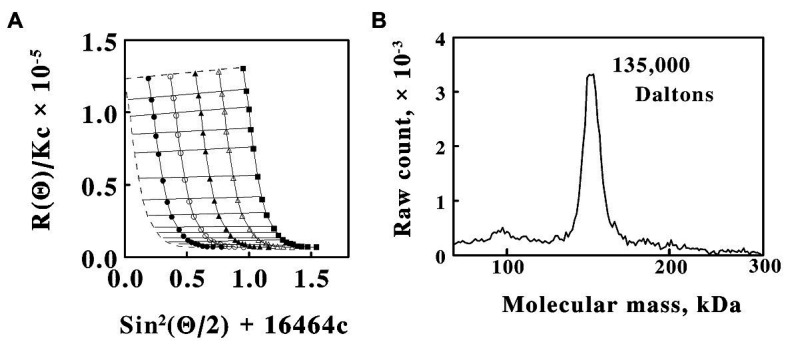
Size analyses of the native cytochrome 579 purified from cell-free extracts of *L. ferriphilum*. **(A)** A Debye plot constructed from static light scattering measurements at different angles and concentrations of purified protein n 0.01 N sulfuric acid. Protein concentrations in μg/ml: 1.15, *closed circles*; 2.31, *open circles*; 3.46, *closed triangles*; 4.62, *open triangles*; and 5.77, *closed squares*. *R*(*Ø*) is the excess Rayleigh ratio, *Ø* is the angle between the incident and the scattered light, *K* is an instrument constant that is determined independently, *c* is the concentration of cytochrome 579, and 16,464 is an arbitrary constant that permitted an appropriate spread of the data for plotting and visualizing the results. Each datum represents the mean of at least eight determinations. The concentration variations and the angular data were fit with first and second order polynomials, respectively. The *dashed lines* represent extrapolation to zero angle (*horizontal line*) and zero concentration (*vertical curve*), respectively. **(B)** Macroion mobility spectrum of purified cytochrome 579 conducted at 320 ng/ml. The spectrum consisted of the accumulated counts of 10 repeat scans of 300 s each.

The apparent molecular mass of native cytochrome 579 in 0.01 N sulfuric acid was determined by Rayleigh light scattering analysis (Wyatt, 1993). Figure 4A shows a Debye plot obtained with five concentrations of cytochrome 579. Extrapolation of the data to zero angle and zero concentration yielded a weight average molecular mass for the native protein of approximately 125,000 Daltons, a value close to that anticipated for an oligomer composed of eight subunits of approximately 16 kDa each. The oligomeric nature of the native protein was also evident from its chromatographic behavior on gel filtration media. Supplementary Figure S2 shows the behavior of native cytochrome 579 when the purified protein was subjected to column chromatography on Sephadex G-75. Sephadex G-75 has an effective fractionation range for globular proteins between 3,000 and 80,000 Da. The native cytochrome 579 eluted from the Sephadex G-75 column in the void volume, thus indicating that the molecular mass of the native protein was, at the very least, in excess of 80,000 Da.

Extrapolation of the data in Figure 4A to zero angle yielded a value for the second virial coefficient (Wyatt, 1993) of cytochrome 579 of 2.3 × 10^−4^ mole ml/g^2^, indicating that the cytochrome and the solvent interacted in a positive manner, an observation consistent with the observed acid solubility and stability of the protein. Extrapolation of the data to zero concentration yielded a curve that was approximately linear near the ordinate intercept. A value of 10.5 nm was calculated from the linear portion of the curve for the root mean square radius of native cytochrome 579. The root mean square radius (also known as the radius of gyration) is the value of the average distance between each of the light-scattering centers (the individual atoms) in the macromolecule and its center of mass as it exists in the acidic aqueous solvent.

The apparent molecular mass of native cytochrome 579 was also determined by macroion mobility spectrometry (macroIMS). MacroIMS is an electrospray ionization technique whereby macromolecules with masses that can exceed 1 million Daltons are dispersed from dilute solution into droplets of 100–200 nl in volume (van Duijn et al., 2005; Carazzone et al., 2008). Desolvation of these droplets generates highly charged particles that then pass through a neutralizing/charge reduction chamber (Scalf et al., 1999) where they are converted into neutral and singly charged nanoparticles. The resulting reduced-charge particles are then separated in the gas phase according to their electrophoretic mobility and quantified with a condensation particle counter. When the macromolecules are prepared in a buffer amenable to electrospray, even noncovalent polypeptide complexes that are captured within the nl droplet can be transferred intact to the gas phase (van den Heuvel and Heck, 2004).

The macroIMS spectrum obtained with purified cytochrome 579 is shown in Figure 4B. A single peak was observed with an apparent molecular mass of 135,000 Daltons. The low protein concentration in the analyte solution, 320 ng/ml, was consistent with the expectation that each 100–200 nl droplet was in the “one analyte per one droplet” operating regime. The 5–6% uncertainty in the absolute accuracy of macromolecular masses as determined by macroIMS measurements (Bacher et al., 2001) meant that these mass results were consistent with those obtained by Rayleigh light scattering analyses.

### Spectral Properties of the Purified Cytochrome 579

**Figure 5:**
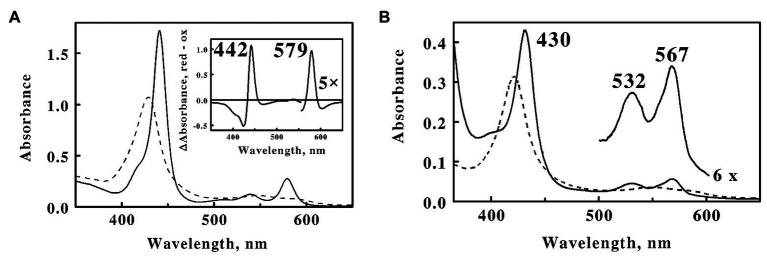
Visible absorbance spectra of cytochrome 579 purified from cell-free extracts of *L. ferriphilum*. **(A)** Spectra of oxidized (*dashed line*) and reduced (*solid line*) cytochrome 579 at 160 μg/ml. Both spectra were determined in 0.01 N sulfuric acid at 25°C. The absorbance spectrum of the reduced cytochrome was determined 10 min after mixing the sample of oxidized cytochrome with an excess of ferrous sulfate. *Inset*, a difference spectrum representing the absolute spectrum of the Fe(II)-reduced cytochrome minus that of the oxidized cytochrome. **(B)** Alkaline pyridine hemochromogen spectra of cytochrome 579 at 45 μg/ml. Spectra: final spectrum of the ferric heme, *dashed curve*; and spectrum of the ferrous heme, *solid curve*.

The absorbance properties of the purified cytochrome are illustrated by the spectra in the visible region shown in Figure 5A. The native oxidized cytochrome exhibited a broad absorbance peak at 426 nm. Electrochemical reduction of the cytochrome by soluble Fe(II) produced new absorbance peaks at 441, 540, and 579 nm. These spectral properties are essentially the same as those reported for the prominent cytochromes in cell-free extracts of *L. ferrooxidans* (Hart et al., 1991) and microbial biofilms collected from an acid mine drainage system at the Richmond Mine (Ram et al., 2005; Singer et al., 2008). The *inset* in Figure 5A is a difference spectrum of the absolute spectrum of the Fe(II)-reduced cytochrome minus that of the oxidized cytochrome. While the peak at 442 nm in the difference spectrum is reminiscent of that of an *a*-type cytochrome, the peak at 579 nm is considerably blue-shifted from that anticipated for a typical *a*-type cytochrome (Smith, 1978). The visible spectrum of the reduced protein did not change when the protein was exposed to saturating concentrations of carbon monoxide, consistent with an overall pattern of hexa-coordinate, low spin ferric, and ferrous hemes.

The alkaline pyridine hemochromes of cytochrome 579 are shown in Figure 5B. The initial spectrum of the ferric protein in alkaline pyridine showed a broad, relatively weak Soret band at 430 nm and a broad visible band at 600 nm (data not shown). This spectrum gradually changed with a half-time of around 6 min to another, more stable, form (*dashed curve*) with a sharp Soret at 418 nm and a weak visible band at 545 nm. The ferrous spectrum (*solid curve*) showed typical absorbance band shapes for a low spin α,β-band ferrous heme, except that the α-band was at the unexpected wavelength of 567 nm. The same ferrous alkaline pyridine hemochromogen spectrum was obtained regardless of whether dithionite was added before, during, or after the slow spontaneous transformation evident in the oxidized alkaline pyridine spectra.

Formation of the reduced pyridine hemochrome spectrum represents a powerful technique used to eliminate those differences in the native absorbance spectra of different heme groups due to the surrounding protein (Bartsch, 1971; Berry and Trumpower, 1987). The unusual α-band at 567 nm in the pyridine hemochrome of cytochrome 579 was significantly higher than those of heme *b* or *c* (557 and 550 nm, respectively; Berry and Trumpower, 1987) or isobacteriochlorin hemes such as siroheme (557 nm; Murphy and Siegel, 1973), yet significantly lower than those in hemes *a* (587 nm) or the *d*-type hemes (618 nm; Berry and Trumpower, 1987; Palmer and Reedijk, 1992). The prosthetic group(s) of cytochrome 579 could not be extracted from the protein by a variety of organic extraction protocols or by thioether bond cleavage reagents typically used to liberate heme *c*, when applied either with or without prior protein denaturation and digestion with proteases. Simultaneous reduction with mercaptoethanol and alkylation in the presence of 4.0 M 4-vinylpyridine (Ozols, 1990) successfully liberated all chromophores from the apoprotein, indicating that the heme may be covalently conjugated to the apoprotein by a novel sulfhydryl bond. Unfortunately, what appeared to be subsequent polymerization of the liberated hemes produced a series of heterogeneous but closely related fractions from which a single purified component has not yet been achieved or characterized. It was evident, however, that cytochrome 579 represents a heme (or hemes) of novel structure that is unique to respiratory iron oxidation in *L. ferriphilum*.

### Electron Transfer Reactions Between Cytochrome 579 and Soluble Iron

The oligomeric nature of the native protein was also evident from kinetic studies on the oxidation and reduction reactions of the purified cytochrome with soluble iron. Purified cytochrome 579 was redox-active with ferric and ferrous sulfate at pH 1.5. Supplementary Figure S3A shows a series of kinetic scans that were acquired when the purified cytochrome 579 was rapidly mixed with 30 mM Fe(II) in a stopped flow spectrophotometer equipped with a rapid scan module. The Soret peak of the cytochrome shifted from 426 nm in the oxidized state to 441 nm in the Fe(II)-reduced state. Concomitantly, an alpha absorbance band appeared with a maximum at 579 nm. The entire set of kinetic data in Supplementary Figure S2A was described by a single exponential function of time (t):

$$\mathrm{absorbance}=B+\left(A-B\right)\;e^{-\mathrm{kt}}$$

where *A* and *B* represent the absolute spectrum of the oxidized and reduced cytochrome, respectively, and *k* is the pseudo-first order rate constant for the absorbance change at all wavelengths within the rapid scan. The value of *k* that provided the best fit of the above equation to the kinetic data in Supplementary Figure S3A was 2.8 ± 0.2 s^−1^. Supplementary Figure S3B shows a single kinetic trace extracted from a three-dimensional plot such as that shown in Supplementary Figure S3A, except that the concentration of soluble ferrous iron in S3B was 60 mM. Like the entire data set at all wavelengths, the increase in absorbance at 579 nm that was concomitant with the reduction of the cytochrome 579 fit a single exponential function of time with a pseudo-first order rate constant to 84 ± 5 s^−1^.

**Figure 6:**
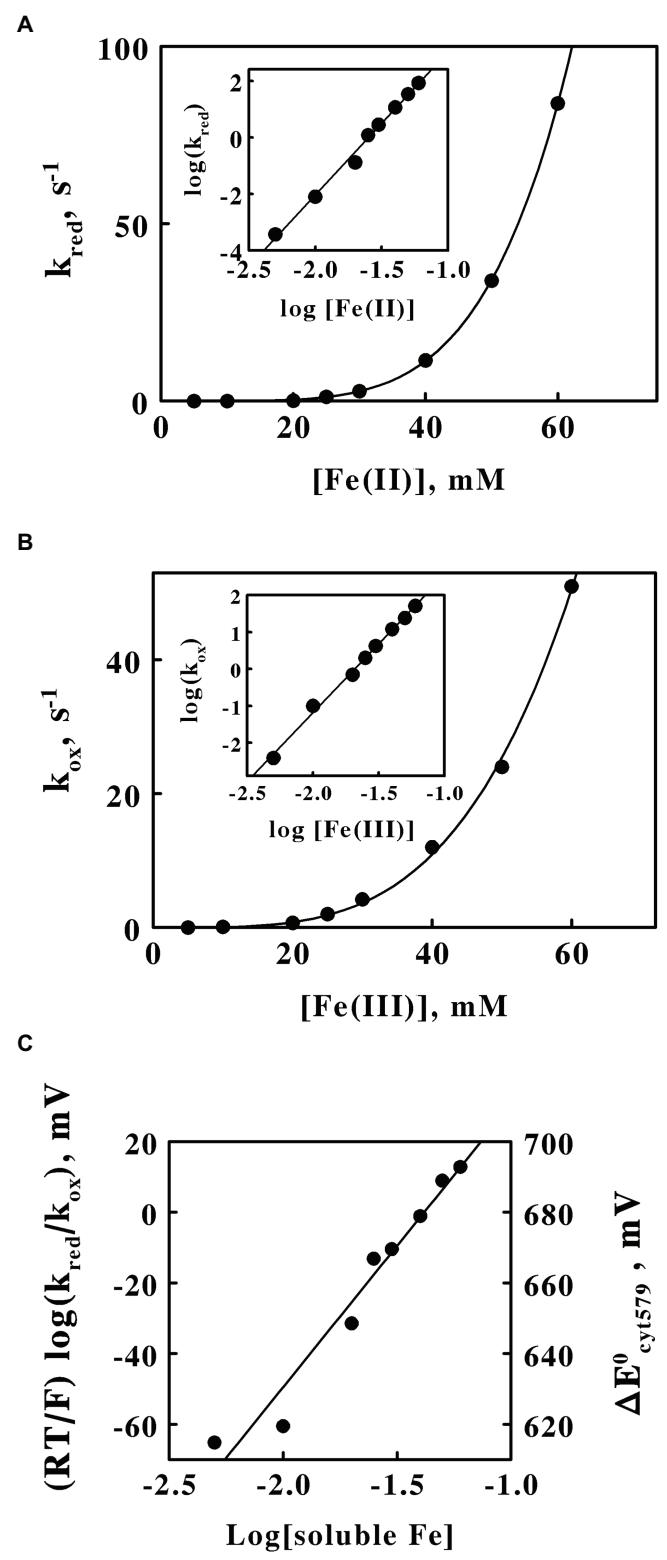
Reactivity of cytochrome 579 with soluble iron at pH 1.5. Absorbance changes were monitored in a stopped flow spectrophotometer when the purified cytochrome was rapidly mixed with a molar excess of soluble iron. **(A)** Dependence of the pseudo-first order rate constant for the reduction of oxidized cytochrome 579 on the concentration of ferrous ions. Final concentrations after mixing: cytochrome 579, 8.6 μM; and sulfuric acid, 0.02 M. The *curve* drawn through the data points was proportional to the concentration of ferrous ions raised to the power of 5.0. *Inset*, the dependence of the logarithm of the pseudo-first order rate constant on the logarithm of the molar concentration of ferrous ions. **(B)** Dependence of the pseudo-first order rate constant for the oxidation of reduced cytochrome 579 on the concentration of ferric ions. Final concentrations after mixing: cytochrome 579, 8.2 μM; and sulfuric acid, 0.02 M. The *curve* drawn through the data points was proportional to the concentration of ferric ions raised to the power of 3.7. *Inset*, the dependence of the logarithm of the pseudo-first order rate constant on the logarithm of the molar concentration of ferric ions. **(C)** Dependence of the logarithm of the ratio of the pseudo-first order rate constants for the reduction and oxidation of the cytochrome on the logarithm of the concentration of soluble iron. The reduction potentials for the purified cytochrome on the right ordinate were obtained using a standard reduction potential for the ferric/ferrous couple of 680 mV in 0.02 M sulfuric acid at pH 1.5.

The dependence of the observed pseudo-first order rate constant for the Fe(II)-dependent reduction of cytochrome 579 on the concentration of Fe(II) is shown in Figure 6A. The concentration of ferrous ions had a profound influence on the value of the rate constant. A mere 12-fold increase in the concentration of the reducing agent (5–60 mM) increased the value of the apparent rate constant over 22,000-fold from 3.7 × 10^−4^ to 84 s^−1^. The *inset* in Figure 6A shows a linear plot of the logarithm of the pseudo-first order rate constant as a function of the logarithm of the soluble ferrous iron concentration. The slope of this log-log plot, 5.0, is effectively the Hill coefficient for the reduction reaction and represents the power to which the concentration of ferrous ion must be raised in order to create a linear correlation among the kinetic data.

Similar results were obtained when analogous kinetic experiments were conducted on the Fe(III)-dependent oxidation of the reduced cytochrome. The dependence of the observed pseudo-first order rate constant for the Fe(III)-dependent oxidation of reduced cytochrome 579 on the concentration of Fe(III) is shown in Figure 6B. The concentration of ferric ions also had a strong influence on the value of the rate constant for oxidation of the reduced cytochrome. The same 12-fold increase in the concentration of the oxidizing agent increased the value of the apparent rate constant 13,000-fold from 3.8 × 10^−3^ to 51 s^−1^. The *inset* in Figure 6B shows a linear plot of the logarithm of the pseudo-first order rate constant as a function of the logarithm of the soluble ferric iron concentration. The slope of this log-log plot, 3.7, is the Hill coefficient for the oxidation reaction and represents the power to which the concentration of ferric ion must be raised in order to create a linear correlation among the oxidation kinetic data.

Figure 6C shows the dependence of the logarithm of the ratio of kinetic rate constants for electron transfer on the logarithm of the concentration of soluble iron. The ratio of the iron-dependent reduction and oxidation rate constants for cytochrome 579 taken from Figures 6A,B, respectively, comprises the equilibrium constant for the following reaction:

Cyt579(III) + Fe(II) ↔ Cyt579(II) + Fe(III)

The rate and equilibrium constants for this electron transfer reaction are related as follows:

$$\left(\mathrm{RT}/\mathrm{nF}\right)\log\left(k_{\mathrm{red}}/k_{\mathrm{ox}}\right)=\Delta E^{0}{}_{\mathrm{Cyt}579}-\Delta E^{0}{}_{\mathrm{Fe}}$$

where *k_red_* and *k_ox_* represent the pseudo-first order rate constants for the iron-dependent reduction and oxidation, respectively, of cytochrome 579, ΔE^0^_Cyt579_ and ΔE^0^_Fe_ represent the standard reduction potentials for cytochrome 579 and soluble iron, respectively, *R* is the gas constant, *T* is the temperature, *F* is Faraday’s constant, and *n* is the number of electrons transferred in the reaction, in this case, one. If the data in Figures 6A,B were simply linear second-order plots, then the resulting logarithm of the ratio of rate constants would be single value that one could use to calculate a single value of ΔE^0^_Cyt579_ from the calculated value of ΔE^0^_Fe_ = 680 mV in 0.02 M sulfuric acid, pH 1.5 (Blake and Shute, 1987; Blake et al., 1991). However, the striking nonlinearity of the kinetic data in Figures 6A,B creates the relationship shown in Figure 6C, where the logarithm of the ratio of kinetic rate constants is itself a linear function of the logarithm of the concentration of soluble iron. This relationship implies that the standard reduction potential of cytochrome 579 is itself a function of iron concentration, which can only happen if soluble iron binds to the octameric cytochrome and thereby influences its electron transfer reactivity and redox potential. At an iron concentration of approximately 40 mM, where *k_red_*/*k_ox_* = 1.0, the reduction potential of cytochrome 579 is equal to that of the soluble iron in sulfuric acid, or 680 mV. At iron concentrations higher than 40 mM, the cytochrome 579 has a higher reduction potential than 680 mV and becomes more oxidizing, while at iron concentrations lower than 40 mM, the cytochrome 579 has a lower reduction potential than 680 mV and becomes less oxidizing. These conclusions are summarized by the corresponding calculated changes in the reduction potentials shown on the *right* ordinate in Figure 6C.

## Discussion

The first notable feature of these results is the unusual absorbance properties of the purified cytochrome. The absorbance maximum at 579 nm in the reduced state is too long for the heme to be a classic *b*- or *c*-type heme protein but too short to be an *a*- or *d*-type heme protein (Palmer and Reedijk, 1992). This observation is particularly interesting because all seven of the homologous cytochromes 579 identified above contain the CXXCH amino acid sequence that is generally acknowledged to represent a signature sequence for heme binding in *c*-type heme proteins. While it is evident from Figure 3A that the heme binding motif was only partially sequenced in the purified protein, the amino acids that surrounded the two difficult-to-measure cysteine residues in the motif were nonetheless identical to those in the other cytochrome 579 homologs. Additional evidence that the heme group associated with cytochrome 579 has a unique structure comes from the unexpected absorbance properties of the heme’s reduced alkaline pyridine hemochromogen spectrum. When heme proteins are exposed to strong base in the presence of high concentrations of a nitrogenous iron ligand like pyridine, the heme is released from the denatured protein and pyridines become the heme’s 5th and 6th axial ligands. The absorbance properties of such hemes are entirely dependent on the structures of the hemes themselves and are no longer influenced by their former native environoments in their respective intact heme proteins (Bartsch, 1971; Berry and Trumpower, 1987). The reduced alkaline pyridine hemochromogen absorbance peaks obtained herein at 567 nm in the absolute spectrum and 568–569 nm in the reduced minus oxidized difference spectrum (data not shown) are well-above those of *b*- and *c*-type hemes (557 and 550 nm, respectively) and well-below that of *a*-type hemes (587 nm; Berry and Trumpower, 1987; Palmer and Reedijk, 1992). The reduced alkaline pyridine hemochromogen spectrum of the cytochrome 579 purified from the community biofilm was reported at 587 nm (Singer et al., 2008), which matches that of an *a*-type heme (Berry and Trumpower, 1987), but does not match that of 567–568 nm reported herein. Of course, the heterogeneous community biofilm could have well-contained relatively higher levels of *a*-type heme proteins.

A cytochrome with a reduced absorbance peak at 572 nm was produced when a recombinant cytochrome *c* from *Thermus thermophilus* was expressed in *Escherichia coli* and heated to 70°C at neutral pH (Fee et al., 2004). Evidence was presented that one of the two vinyl groups in the heme was oxidized to a formyl group. The standard reduction potential of this recombinant cytochrome 572 was 340 mV, approximately 120 mV more positive than that of the recombinant cytochrome *c* with two intact vinyl groups. The reduced alkaline pyridine hemochromogen spectrum of this recombinant, heated cytochrome 572 had an absorbance maximum at 581 nm. However, the peak absorbance of the reduced alkaline pyridine hemochromogen of the native cytochrome 572 purified from the microbial community biofilm was given as 568 nm (Singer et al., 2008), which actually matches that reported herein for cytochrome 579 purified from the type strain of *L. ferriphilum*. It is evident that more experiments are required to clear up these discrepancies. In the meantime, it has become evident that cytochrome 579 represents the product of a novel heme that may potentially interact with the CXXCH sequence motif in a novel manner depending on the nature of any structural modifications in the heme group which have yet to be fully established.

The second notable feature of these results is the realization that the genetic sequences of each of the seven strains of *L. ferriphilum* that are available at the present time only contain the capacity to express two homologs or isozymes per strain of all the seven possible cytochromes 579. Further, each of the two homologs present in each strain is expressed in conjunction with a different signal peptide. The influence, if any, of these two different signal peptides on the eventual extracytoplasmic localization of the respective cytochromes 579 is unknown. There are literally hundreds of different signal peptides tentatively identified in prokaryotes. For example, careful proteogenomic studies confirmed 156 out of the 337 signal peptides predicted by version 4.0 of SignalP in the complete genome of *E. coli* strain K-12 (Ivankov et al., 2013). Far fewer, if any, putative signal peptides have been confirmed by actual experimentation in lesser-studied organisms like *L. ferriphilum*. Is one homolog of cytochrome 579 destined for the periplasm, while the other homolog is destined for the outer membrane? We simply do not know. What we do hypothesize is that both homologs were co-purified from acidic cell-free extracts of the type strain cultured herein on soluble ferrous ions. This hypothesis is based on the following two observations: (i) the deconvoluted positive-ion electrospray mass spectrum of the purified protein shown in Figure 2B provided evidence of two polypeptides that differed by only 125 Da; and (ii) the protein bands in Figure 2A that represented the purified cytochrome 579 at different concentrations were sufficiently diffuse so as to suggest the presence of two polypeptides with molecular masses that were not separable by this technique (16,015 vs. 16,140 Da). Because all of the homologs featured in Supplementary Figure S1 are highly similar with only minor, generally conservative, amino acid differences, it is perhaps not surprising that the kinetic studies conducted herein and elsewhere (Blake and White, 2020) exhibited no evidence of heterogeneity in the purified cytochrome’s functional behavior. Whether the purified cytochrome studied herein is a mixture of two homologs, or a mixture of one homolog with varying degrees of post-translational modification, or some combination of both, the fact remains that the purified cytochrome behaves as would a single homogeneous protein.

The sequence of the first 49 amino acids that was obtained using the purified cytochrome 579 was not identical to those of the proteins with accession numbers WP_036080593.1 and WP_014961757.1, the two homologs of cytochrome 579 expected to be expressed by the type strain (Figure 3C). Likewise, the molecular masses of the two protein subunits presented in Figure 2B did not exactly match those expected from the two homologs in the type strain minus the masses of their respective signal peptides and the associated heme groups. However, it should be noted that others have reported posttranslational modifications and sequence variations of cytochromes 579 present in *Leptospirillum*-dominated biofilms in different stages of development (Singer et al., 2010). One can also note that the type strain of *L. ferriphilum* was received in this laboratory from the DSMZ over 20 years ago as an active culture, and that the culture has been maintained by continually passaging the active planktonic culture the entire time. Consequently, it is entirely possible that slight genetic drift has occurred in the cytochromes 579 during that period compared with the cytochromes 579 present in “identical” strains that have been similarly maintained for long periods of time in other laboratories.

The third notable feature of these results is the observation, by two entirely different analytical methods, that the native cytochrome 579 appeared to be an octameric protein over nearly a 20-fold range in protein concentrations. At the lower end of this range, 320 ng/ml or 20 nM in subunits, macroion mobility spectrometry could easily detect sub-nanomolar concentrations of individual subunits or oligomeric proteins of less than eight subunits. This laboratory previously quantified monoclonal antibodies whose antigen binding sites were occupied with 0, 1, or 2 protein antigens when the total antibody concentration was 5 nM, or 10 nM in binding sites (Blake and Blake, 2012). There were no hints of lower-weight protein intermediates in the spectrum shown in Figure 4B. At the higher end of this range, 5.77 μg/ml or 360 nM in subunits, static light scattering measurements could easily detect polymerization of the cytochrome 579 to aggregates greater than eight subunits. If further aggregation had occurred, the weight-average molecular mass on the ordinate intercept would simply have been higher than that shown in Figure 4A. The reasons why the other purified cytochromes 579 do not appear to be oligomeric (Hart et al., 1991; Singer et al., 2008) are not known.

What are the advantages, if any, if cytochromes 579 form octamers in the acidic periplasm of *L. ferriphilum*? There is no evidence that bulk ferrous ions enter the neutrophilic cytoplasm when *L. ferriphilum* or related species respire aerobically on their limited range of inorganic electron donors. Thus, whether the electron donor is soluble iron or an insoluble iron-containing sulfide mineral, respiratory electrons must be conducted from the periphery of the Gram-negative organism across the periplasm to a terminal oxidase embedded in the plasma membrane. An impressive volume of work has been reported on prokaryotes that conduct dissimilatory reduction of extracellular soluble and insoluble iron oxides under anaerobic or microaerophilic conditions (Lovley et al., 2004; Weber et al., 2006; Paquete and Louro, 2010, 2014). A comprehensive *in silico* survey of 594 complete prokaryotic genomes identified 1,659 multiheme *c*-type cytochromes in 258 of the organisms (Sharma et al., 2010). A clear subset of these 258 organisms had the capacity to express many different multi-heme *c*-type cytochromes into their periplasm and/or their outer membranes, ostensibly to facilitate the required dissimilatory electron transfer reactions to extracellular soluble and insoluble acceptors. Although *L. ferriphilum* has not been shown to conduct dissimilatory electron transfer to extracellular acceptors (Weber et al., 2006), its aerobic energy metabolism is totally dependent on electron flow in the opposite direction from extracellular electron donors to the cytoplasmic membrane. Whether an organism expresses one large multiheme protein with 6–10 hemes or eight smaller monoheme proteins that form a stable octamer would seem to make little difference in the final product. We hypothesize that this eight-heme octamer functions to facilitate electron flow across the periplasm of *L. ferriphilum* during aerobic respiration on extracellular reduced iron.

The oligomeric nature of the native cytochrome 579 was also evident from the fourth notable feature of these results, the extraordinary dependencies of the rate constants for the iron-dependent reduction and oxidation of the cytochrome on the concentrations of soluble iron. The strong kinetic synergy in the reduction and oxidation of the oligomeric protein suggested that there is facile communication among the redox states of the eight heme groups, just as is hypothesized for the individual hemes in the decaheme cytochromes *c* in *Shewanella* (Paquete and Louro, 2010, 2014). Although the rates of the reduction of the purified cytochrome at Fe(II) concentrations in excess of 30 mM were clearly sufficiently rapid to be of physiological significance, the corresponding rates of reduction at sub-mM concentrations of Fe(II), conditions that are more likely to be of physiological significance to the intact organism, were far too slow to account for the overall rate of oxygen consumption by these organisms. Others have noted that the iron-dependent reduction of cytochrome 579 is faster and more complete in proteins extracted from early-stage biofilms as opposed to proteins taken from more mature biofilms (Singer et al., 2008). It has even been proposed that the slightly different cytochrome 579 that is expressed in early-stage biofilms could serve as an alternative initial oxidant of soluble iron, along with the cytochrome 572 in the outer membrane (Singer et al., 2010). Do planktonic *L. ferriphilum* behave more like organisms in early-stage biofilms than do those in later stages of biofilm development?

Of course, conditions experienced by the cytochrome 579 in the crowded, gel-like environment of the periplasm may not have been duplicated by the *in vitro* kinetic experiments conducted on purified proteins in dilute solutions. Rather, the functional properties of an intact electron transport chain that consists of the sequential transfer of electrons from iron to cytochrome 572 to cytochrome 579 to a terminal oxidase in the plasma membrane are inferred from observations on isolated biomolecules in dilute solutions. This laboratory has utilized an integrating cavity absorption meter to monitor absorbance changes in intact microorganisms as they respire aerobically on reduced iron (Blake and Griff, 2012; Li et al., 2015; Blake et al., 2016; Blake and White, 2020). Kinetic studies conducted on intact *L. ferrooxidans* (Blake and Griff, 2012) and intact *L. ferriphilum* (Blake and White, 2020) were both consistent with the hypothesis that reduced cytochrome 579 was an obligatory intermediate in the aerobic iron respiratory chain of both organisms. It is evident that the direct and accurate observation of absorbance changes *in situ* in intact organisms can be a useful complement to traditional reductionist approaches and to recent advances in proteomic and transcriptomic studies. In the case of *L. ferriphilum*, the ferrous iron-dependent reduction of the cytochrome 579 in the intact organism was complete within the 1 s mixing dead time of the instrument, even when the soluble ferrous concentration was as low as 50 μM (Blake and White, 2020). It was evident that the rate of the iron-dependent reduction of the cytochrome 579 *in situ* was far more rapid than was the corresponding rate of reduction of the purified protein *in vitro* under the same solution conditions. These contradictory observations constitute a cautionary example where a functional behavior that was monitored *in vitro* did not accurately reflect the analogous functional behavior that was monitored *in situ*.

## Data Availability Statement

The raw data supporting the conclusions of this article will be made available by the authors, without undue reservation.

## Author Contributions

RB wrote the manuscript and directed the project, and also collected and interpreted the stopped-flow kinetic data. JS directed the N-terminal amino acid sequencing and the electrospray mass spectrometry on the native cytochrome. RT conducted the alkaline pyridine hemochromogen studies. RW constructed the phylogenetic tree of the different homologs of cytochrome 579. All authors contributed to the article and approved the submitted version.

### Conflict of Interest

The authors declare that the research was conducted in the absence of any commercial or financial relationships that could be construed as a potential conflict of interest.

## Abbreviations

SDS-PAGE: Sodium dodecyl sulfate polyacrylamide gel electrophoresis
NCBI: National Center for Biotechnology Information
DSMZ: Deutsche Sammlung von Mikrooranismen und Zellkultuen GmbH
BLAST: Basic Local Alignment Search Tool
BLASTp: BLAST search of protein databases using a protein query
tBLASTn: BLAST search of nucleotide databases using a protein query translated into its corresponding nucleic acid sequence
