# Supplementary material for: Homogeneous Cytochrome 579 Is an Octamer That Reacts Too Slowly With Soluble Iron to Be the Initial Iron Oxidase in the Respiratory Chain of Leptospirillum ferriphilum
Source: Front Microbiol. 2021 May 3;12:673066. doi: 10.3389/fmicb.2021.673066 (PMC8126622; doi:10.3389/fmicb.2021.673066)

Figure S1. Alignment of the entire amino acid sequences of seven homologs of cytochrome 579 obtained from tBLASTn searches of the genomes of purified strains of *L. ferriphilum* that are contained in the NCBI database. The protein with the accession number of WP_168058047.1 is a monoheme *c*-type cytochrome in the genome of *Candidatus Manganitrophus noduliformans* that was selected as an appropriate outgroup for cytochrome 579.

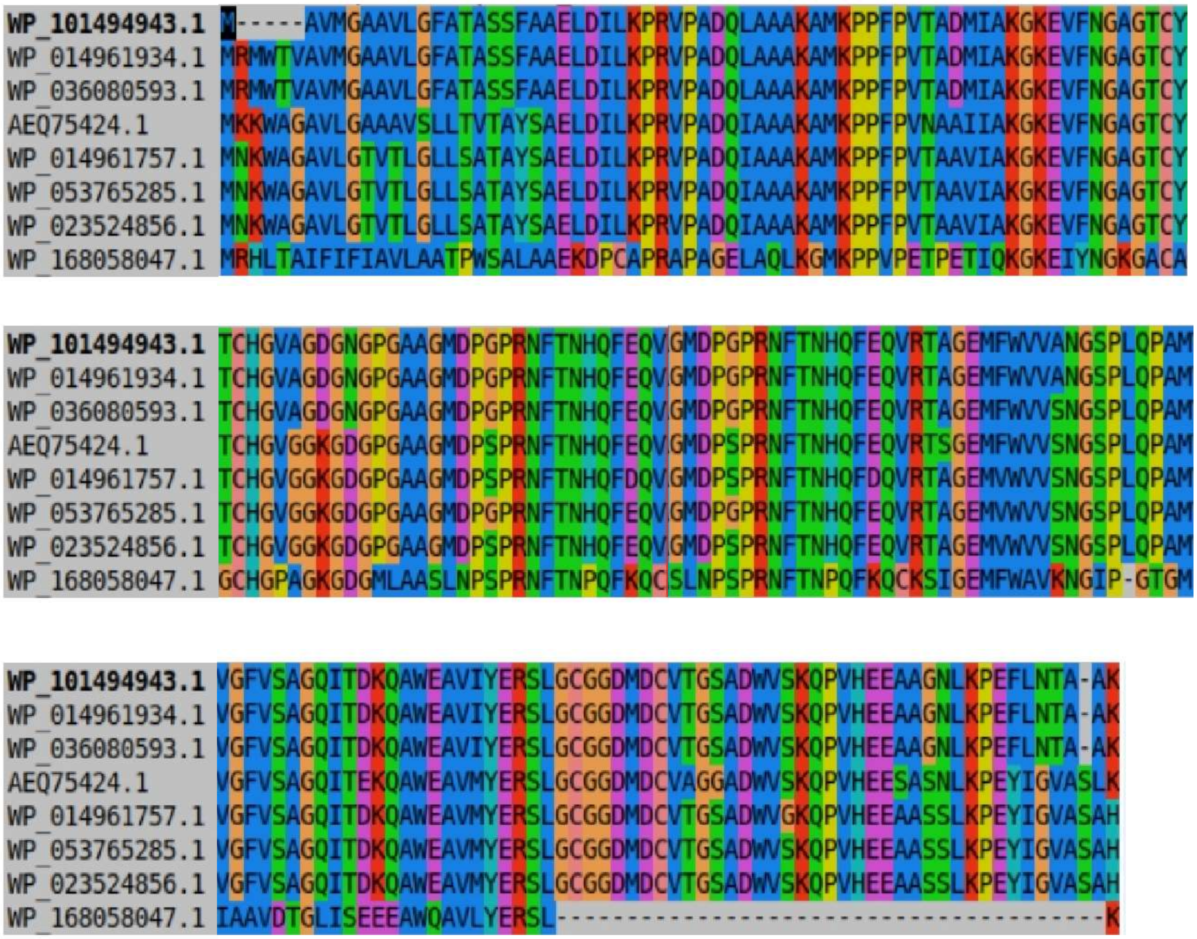

Figure S2. Gel filtration chromatography of purified cytochrome 579 using Sephadex G-75. Hydrated Sephadex G-75 was packed into a column 25 cm long and 2 cm in diameter and equilibrated with two column volumes of 0.001 N sulfuric acid. Two ml that contained a total of 320 µg of reduced cytochrome 579 in 0.02 M sulfuric acid was applied to the top of the resin, and the chromatogram was subsequently developed with 0.001 N sulfuric acid. Two ml fractions were collected at a flow rate of 1.5 ml/min, and the appearance of reduced cytochrome 579 was monitored by measuring the absorbance of the contents of each fraction at 440 nm. The positions of the void (V_0) and total liquid volumes (V_t) of the column were determined in independent gel filtration studies using the same column by monitoring the appearance of Blue Dextran (λ=610 nm, mass = 2000 kDa) and ferricyanide (λ=420 nm, mass = 212 Da), respectively.

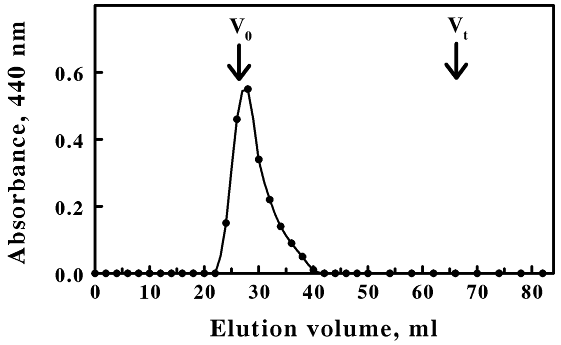

Figure S3. Reactivity of cytochrome 579 with soluble iron at pH 1.5. ***A***, kinetic scans from 370 to 600 nm when purified cytochrome 579 was mixed rapidly with an excess of soluble ferrous ions. The reaction was monitored at 35° C in a dual-beam stopped flow spectrophotometer equipped with a rapid scan module. A 230-nm scan was taken every millisecond for 1 s; every 50th scan is presented. Final concentrations in the observation cell after mixing were: cytochrome 579, 8.6 µM; ferrous ions, 30 mM; and sulfuric acid, 0.2 M. The pH was 1.5. The reference solution in the dual-beam instrument contained all components except the cytochrome. ***B***, a single kinetic trace extracted from a three-dimensional plot such as that shown in ***A***, except that the concentration of soluble ferrous iron in ***B*** was 60 mM. Like the entire data set at all wavelengths, the increase in absorbance at 579 nm that was concomitant with the reduction of the cytochrome 579 fit a single exponential function of time with a pseudo-first order rate constant to 84 ± 5 s^{-1}.

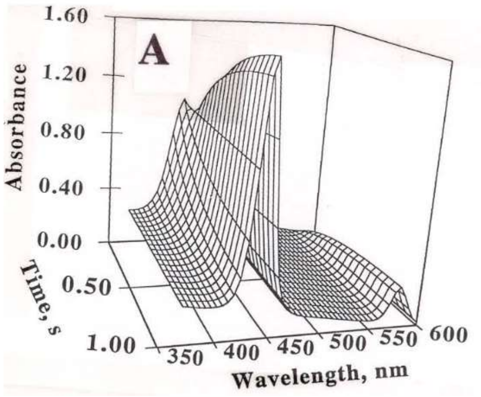

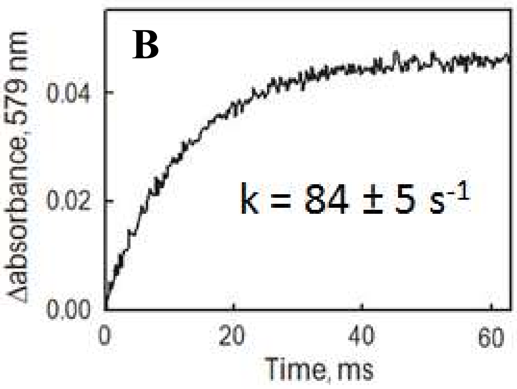

Supplement: Supplementary file 1 [file Data_Sheet_1.PDF]
